# Calf circumference has a positive correlation with physical performance among community-dwelling middle-aged, older women

**DOI:** 10.3389/fpubh.2022.1038491

**Published:** 2022-12-09

**Authors:** Po-Chun Wang, Wei-Chung Yeh, Yi-Wen Tsai, Jau-Yuan Chen

**Affiliations:** ^1^Department of Family Medicine, Linkou Chang Gung Memorial Hospital, Taoyuan, Taiwan; ^2^Department of Family Medicine, Chang Gung Memorial Hospital, Keelung, Taiwan; ^3^Department of Family Medicine, New Taipei Municipal Tu-Cheng Hospital (Built and Operated by Chang Gung Medical Foundation), New Taipei City, Taiwan; ^4^College of Medicine, Chang-Gung University, Taoyuan, Taiwan

**Keywords:** calf circumference, community-based, sex-based differences, sarcopenia and frailty, physical performance, middle-aged and elderly

## Abstract

**Introduction:**

Sarcopenia and frailty are well-known public health problems in middle-aged and older people. Calf circumference (CC) is a representative anthropometric index that may be useful for screening sarcopenia. Physical performance, assessed by hand grip strength and gait speed, measures sarcopenia and frailty. This community-based, cross-sectional study was conducted in Guishan District, Taoyuan City, between April and October 2017 to investigate the relationship between CC and physical performance among community-dwelling middle-aged, older people in Taiwan and to evaluate potential sex differences. CC tends to be an efficient predictor of physical performance in community health screenings and outpatient clinics for community health examinations, where there is limited time for surveys.

**Methods:**

A total of 1,308 volunteers aged 50–85 were recruited. Volunteers who declined to participate, those with recent cardiovascular disease, and those with an inability to complete an interview, physical performance examinations, and body composition measurements were excluded from the study. A total of 828 participants were enrolled in this study (237 men and 591 women). The statistical methods applied in this study were the Mann–Whitney *U*-test, independent two-sample *t*-test, Chi-square test, and multivariate logistic regression models.

**Result and discussion:**

Significant differences were observed in age, waist circumference, appendicular skeletal mass index, calf circumference, hand grip strength, and income between men and women. No significant differences were observed between the men and women regarding body mass index, gait speed, exercise habits, or underlying disorders of diabetes mellitus, hypertension, or hyperlipidemia. Comparing across three different CC tertiles, we discovered significant differences in age, body mass index, waist circumference, appendicular skeletal muscle index, gait speed, and hand grip strength in both men and women. On multivariate logistic regression, after adjusting for age, appendicular skeletal mass index, body mass index, exercise habits, income levels, and CC were positively correlated with physical performance as measured by both gait speed (β = 0.15, *p* = 0.01) and hand grip strength (β = 0.25, *p* < 0.001) in women, compared to only hand grip strength (β = 0.41, *p* < 0.001) in men. Lower calf circumference is an independent risk factor for poor physical performance, especially among women.

## Introduction

Loss of muscle mass is associated with physical inactivity, decreased mobility, and slow gait speed. Muscle loss may result in decreased physical performance, an increased risk of accidental falls, and a sedentary lifestyle. Sarcopenia is a major public health concern for middle-aged and older people. It has been correlated with adverse outcomes, including disability, poor quality of life, and increased mortality risk (1, 2). Even in healthy older people, there is a significant rate of sarcopenia (3). According to a recent systematic review and meta-analysis, the overall estimates of sarcopenia prevalence in different regions globally are 10% (95% CI: 8–12%) in men and 10% (95% CI: 8–13%) in women (4). Xin et al. (5) performed a meta-analysis and found that the pooled prevalence of sarcopenia in older Chinese adults was 14% (95% CI: 11–18%). Its prevalence was higher in Chinese women than in men (15 vs. 14%). Early screening and diagnosis may reduce adverse outcomes.

Anthropometric measurements can be useful tools for the clinical assessment of sarcopenia in older people. Currently, bioelectrical impedance analysis (BIA) and dual-energy X-ray absorptiometry (DXA) are tools for measuring body composition and sarcopenia assessment (6, 7). BIA estimates muscle mass based on whole-body electrical conductivity (7–11), while DXA includes a pencil-beam or fan-beam densitometer to determine muscle quantity non-invasively (7, 12–14). However, their utilization among older subjects has limitations: BIA is more invasive, especially for those with an implantable pacemaker (15), whereas DXA may be costly and unavailable in the community (8).

The association between anthropometric indices and physical fitness performance has also been studied by several other people. Mamphwe et al. (16) reported that calf circumference (CC) might be a useful predictor of both muscle strength and physical performance (hand grip strength and walking speed) in middle-aged and older black men and, to a limited extent, in middle-aged and older black women. Silva et al. (17) showed that a lower CC was associated with worse hand grip strength and balance in older Brazilians aged >60 years. Soh and Won (18) found that poor physical performance was correlated with body fat composition in women, while fat-free mass was positively associated with better physical performance in men. As sarcopenia is associated with a significantly higher risk of mortality, independent of population and sarcopenia definition, there is a need for effective screening tools and early diagnosis for all people (19). Therefore, the relationship between physical fitness performance and anthropometric indices (particularly CC) warrants further research.

Calf circumference is a representative anthropometric index for sarcopenia screening, and hand grip strength and gait speed play essential roles in the diagnosis of sarcopenia and frailty (6, 7). Several definitions of sarcopenia have been proposed, such as those of the European Working Group on Sarcopenia in Older People (EWGSOP and EWGSOP2) (7) and the Asian Working Group for Sarcopenia (AWGS) (6). The cutoff values of CC, hand grip strength, and gait speed were defined differently due to different references to people of different ethnicities. In addition, to achieve a frailty diagnosis, three of the following five components must be satisfied: low energy, low physical activity level, unintentional weight loss, low grip strength, and slow walking speed (20).

There are limited facilities for surveys in community health screenings and outpatient clinics for community health examinations (21, 22); thus, efficient tools are necessary for assessment. Whether CC is a representative anthropometric index correlated with physical performance (hand grip strength and gait speed) is unknown and should be studied further. In addition, a few studies evaluated sex-based differences in the correlations between CC and physical performance (18). A few studies were conducted on the Taiwanese population (23). However, this study aimed to explore and discuss the correlations between CC and physical performance, including sex-based differences, among the community-dwelling middle-aged and older population in Taiwan.

## Methods

### Study design

This is a community-based and cross-sectional study. The participants in this study were recruited from a community health promotion project registered at Chang Gung Memorial Hospital-Linkou Branch, one of the medical centers in northern Taiwan, between April and October 2017. The participants were a subgroup within a previously published study (24).

### Participants and settings

Initially, we started the study by continuously recruiting 1,308 volunteers through poster promotion or notification from the community office from 27 randomized clusters of 32 villages in Guishan District, Taoyuan City. Each participant completed a structured questionnaire during a face-to-face interview with trained research assistants. The questionnaire included personal information, past medical history, self-reported medical or surgical conditions, and current medication use.

### Inclusion and exclusion criteria

The inclusion criteria were (1) subjects aged between 50 and 85 years and (2) those having lived in the same district of residence for more than 6 months. The exclusion criteria were (1) failure to complete body composition analysis (*n* = 14), (2) an inability to communicate adequately to complete an interview (*n* = 10), (3) functional dependency such as inability to walk 6 m (*n* = 8), (4) recent diagnosis of cardiovascular disease (CVD) (*n* = 8), and (5) refusal to participate (*n* = 440). Thus, 828 participants (237 men and 591 women) were enrolled in the analysis.

### Ethical report

This study followed the guidelines for research with human beings in the Helsinki Declaration, as amended in 2013. The data were approved by the Chang Gung Medical Foundation Institutional Review Board, and consent was obtained from all participants before enrollment. We explained the study process well before anthropometric measurements and physical performance exams. No fall accidents or acute cardiovascular complications were observed during the study.

### Data collection

Data collection included body mass index (BMI), waist circumference, body composition, gait speed, hand grip strength, exercise habits, income, and past medical history. BMI was calculated as weight (kg) divided by height squared (m^2^). Waist circumference was measured midway between the lowest ribs and iliac crest, as recommended by the World Health Organization and the International Diabetes Federation (25). Body composition was assessed using the TANITA body composition analyzer BC-418 to establish appendicular skeletal muscle mass (ASM) and appendicular skeletal mass index (ASMI). ASM was defined as the sum of the muscle mass of the four limbs, and ASMI was calculated as ASM/height^2^ (m^2^), according to AWGS (6) and EWGSOP (7). In addition to past medical history, questionnaires on personal information were also used to evaluate baseline exercise habits and income. Participants' exercise habits were defined as infrequent unless they engaged in it three times per week and for more than 30 mins each time. The participants were divided into three groups according to monthly income level: group 1 (<20,000 NTD), group 2 (20,000–40,000 NTD), and Group 3 (>40,000 NTD). Underlying diseases considered included diabetes mellitus (DM), hypertension (HTN), and hyperlipidemia.

### Assessment of calf circumference

Anthropometric measurements were performed using non-elastic, flexible plastic tape. Calf circumference (CC) was measured on the left leg (or the right leg for left-handed persons) in a sitting position with the knee and the ankle at a right angle and the feet resting on the floor. CC was measured at the point of the greatest circumference. The subcutaneous tissues were not compressed. The CC cutoff values in **Table 2** were calculated using tertiles.

### Assessment of muscle strength and physical performance

We measured the time taken to walk 6 m three times and calculated the average gait speed. Hand grip strength in both hands was measured two times with standard positioning's recommended by AWGS 2019, standing with full elbow extension for the Takei T.K.K.5401 GRIP-D handgrip dynamometer (Takei Scientific Instruments Co., Ltd., Tokyo, Japan), and the highest measurement was recorded (6).

### Statistical analysis

The quantitative variables used were mean standard deviation (SD) for continuous variables and number (%) for categorical variables. Continuous variables with non-normal distributions are shown as medians (interquartile ranges), and *p*-values were calculated using the Mann–Whitney *U*-test. Continuous variables were calculated as *p*-values using an independent two-sample *t*-test. The chi-square test was used to calculate *p*-values for categorical variables. Multivariate logistic regression models were used to examine the relationship between physical performance and CC. All statistical analyses were conducted using IBM Statistical Product and Service Solutions Statistics for Windows (version 19.0; IBM Corp., Armonk, NY, USA). Statistical significance was considered at a *p* < 0.05, which was corrected by the false discovery rate (FDR).

## Results

A total of 828 participants, both men and women, aged 50–85 years (28.62% men, with a response rate of 66.36%), were enrolled in this study. The study population was divided into two groups by sex, as shown in Table 1. Of the 828 participants, the average hand grip strength in men and women was 36.50 ± 8.21 and 23.77 ± 8.21 kg, respectively (*p* < 0.001). The average gait speed in men and women was 1.44 ± 0.27 and 1.43 ± 0.24 m/s (*p* = 0.45), respectively. Age, waist circumference, ASMI, income, CC, and handgrip strength were significantly higher in men than in women. No significant difference was observed between men and women regarding BMI, gait speed, exercise habits, underlying DM, or cardiovascular disease.

**Table 1:** Baseline characteristics of participants by sex.

**Variables**	**Total (*n* = 828)**	**Men (*n* = 237)**	**Women (*n* = 591)**	***P*-value**
Age (years)^†^	64.88 ± 7.99	66.80 ± 8.83	64.11 ± 7.50	<0.001^‡^
BMI (kg/m^2^)^†^	24.50 ± 3.51	24.54 ± 3.07	24.48 ± 3.68	0.84
Waist circumference (cm)	85.08 ± 10.07	88.85 ± 8.81	83.57 ± 10.15	<0.001^‡^
ASMI (kg/m^2^)^†^	7.43 ± 1.18	8.86 ± 1.03	6.85 ± 0.62	<0.001^‡^
Calf circumference (cm)	34.77 ± 2.81	35.59 ± 2.62	34.44 ± 2.82	<0.001^‡^
Hand grip strength (kg)^†^	27.41 ± 8.23	36.50 ± 8.21	23.77 ± 4.63	<0.001^‡^
Gait speed (m/s)^†^	1.43 ± 0.25	1.44 ± 0.27	1.43 ± 0.24	0.45
Exercise habit *n*(%)	602(72.71)	173(73)	429(72.59)	0.91
Income (NTD) *n*(%)				<0.001^‡^
<20,000	626(75.60)	143(60.34)	483(81.73)	
20,001–40,000	133(16.06)	52(21.94)	81(13.71)	
>40,000	69(8.33)	42(17.72)	27(4.57)	
HTN *n*(%)	265(32.00)	79(33.33)	186(31.47)	0.60
DM *n*(%)	119(13.37)	42(17.72)	77(13.03)	0.08
Hyperlipidemia *n*(%)	111(13.41)	28(11.81)	83(14.04)	0.39

Clinical characteristics are expressed as mean ± SD values for continuous variables and n (%) for categorical variables.

^†^Continuous variables with non-normal distributions are shown as median (interquartile range). P-values were derived from the independent two-sample t-test and Mann–Whitney U-test for continuous variables and the chi-square test for categorical variables.

^‡^*P* < (0.05/16).

BMI, body mass index; ASMI, appendicular skeletal muscle index; DM, diabetes mellitus; HTN, hypertension.

According to their general characteristics, the study population was categorized into three different CC tertiles in Tables 2A,B. In both sexes, CC tertiles were negatively correlated with age and positively correlated with BMI, waist circumference, and ASMI. Regarding physical performance, handgrip strength and gait speed were positively correlated with CC tertiles in both sexes.

**Table 2A:** Baseline characteristics of male participants according to tertile increment of calf circumference.

**Variables**	**Men**			***P*-value**
	**Calf circumference**		
	**Tertile 1**	**Tertile 2**	**Tertile 3**	
	**(*n* = 79)**	**(*n* = 79)**	**(*n* = 79)**	
	**(<34.50)**	**(34.50–36.75)**	**(>36.75)**	
Age (years)	68.99 ± 8.41	67.20 ± 9.24	64.22 ± 8.25	0.003^‡^
BMI (kg/m^2^)	22.30 ± 2.10	24.45 ± 2.53	26.87 ± 2.67	<0.001^‡^
Waist circumference (cm)	83.74 ± 6.83	88.23 ± 7.32	94.57 ± 8.65	<0.001^‡^
ASMI (kg/m^2^)	8.02 ± 0.73	8.86 ± 0.81	9.70 ± 0.76	<0.001^‡^
Exercise habit *n*(%)	58(73.42)	50(63.29)	65(82.28)	0.03*
Income (NTD) *n*(%)				0.03*
<20,000	51(64.56)	51(64.56)	41(51.90)	
20,001–40,000	21(26.58)	15(18.99)	16(20.25)	
>40,000	7(8.86)	13(16.46)	22(27.85)	
HTN *n*(%)	25(31.65)	21(26.58)	33(41.77)	0.12
DM *n*(%)	16(20.25)	15(18.99)	11(13.92)	0.54
Hyperlipidemia *n*(%)	6(7.59)	7(8.86)	15(18.99)	0.05
Hand grip strength (kg)	32.89 ± 7.78	37.12 ± 8.06	39.50 ± 7.48	<0.001^‡^
Gait speed (m/s)	1.41 ± 0.28	1.41 ± 0.28	1.51 ± 0.25	0.04*

Clinical characteristics are expressed as mean ± SD values for continuous variables and as n (%) for categorical variables.

P-values were derived from the independent two-sample t-test for continuous variables and from the chi-square test for categorical variables.

*P < 0.05;^‡^*P* < (0.05/16).

BMI, body mass index; ASMI, appendicular skeletal muscle index; DM, diabetes mellitus; HTN, hypertension.

**Table 2B:** Baseline characteristics of female participants according to tertile increment of calf circumference.

**Variables**	**Women**			***P*-value**
	**Calf circumference**			
	**Tertile 1**	**Tertile 2**	**Tertile 3**	
	**(*n* = 197)**	**(*n* = 209)**	**(*n* = 185)**	
	**(<33.20)**	**(33.20-35.50)**	**(>35.50)**	
Age (years)	65.17 ± 7.61	64.45 ± 7.43	62.60 ± 7.25	0.003^‡^
BMI (kg/m^2^)	21.90 ± 2.65	24.32 ± 2.67	27.43 ± 3.45	<0.001^‡^
Waist circumference (cm)	77.66 ± 9.05	83.48 ± 8.50	89.98 ± 9.09	<0.001^‡^
ASMI (kg/m^2^)	6.45 ± 0.48	6.84 ± 0.49	7.30 ± 0.60	<0.001^‡^
Exercise habit *n*(%)	152(77.16)	154(73.68)	123(66.49)	0.06
Income (NTD) *n*(%)				0.26
<20,000	170(86.29)	168(80.38)	145(78.38)	
20,001–40,000	21(10.66)	32(15.31)	28(15.14)	
>40,000	6(3.05)	9(4.31)	12(6.49)	
HTN *n*(%)	48(24.37)	66(31.58)	72(38.92)	0.01*
DM *n*(%)	25(12.69)	30(14.35)	22(11.89)	0.76
Hyperlipidemia *n*(%)	22(11.17)	37(17.70)	24(12.97)	0.15
Hand grip strength (kg)	22.26 ± 4.39	24.03 ± 4.00	25.09 ± 5.09	<0.001^‡^
Gait speed (m/s)	1.39 ± 0.26	1.44 ± 0.23	1.45 ± 0.23	0.04*

Clinical characteristics are expressed as mean ± SD values for continuous variables and as n (%) for categorical variables.

P-values were derived from the independent two-sample t-test for continuous variables and from the chi-square test for categorical variables.

*P < 0.05.

^‡^*P* < (0.05/16).

BMI, body mass index; ASMI, appendicular skeletal muscle index; DM, diabetes mellitus; HTN, hypertension.

Exercise habits and income were positively correlated with CC tertiles in men, while HTN was positively correlated with CC tertiles in women. No significant association with CC was found for the underlying medical conditions of DM or hyperlipidemia in either sex.

Table 3 presents the results of the multivariate linear regression analysis. After adjusting for suspected confounding factors according to previous studies in Model 3 (age, ASMI, BMI, exercise habits, and income), CC was positively correlated with both gait speed (β = 0.15, *p* < 0.01) and hand grip strength (β = 0.25, *p* < 0.001) in women, while it was only positively correlated with hand grip strength (β = 0.41, *p* < 0.001) in men.

**Table 3:** Multivariate linear regression analyses of physical performance by calf circumference, presented with gender difference.

	**Gait speed (m/s)**		**Hand grip strength (kg)**	
	**β**	***P*-value**	**β**	***P*-value**
Men				
Calf circumference				
Model 1	0.19	0.003^‡^	0.42	<0.001^‡^
Model 2	0.47	0.64	0.39	<0.001^‡^
Model 3	0.04	0.67	0.41	<0.001^‡^
Women				
Calf circumference				
Model 1	0.09	0.02^‡^	0.29	<0.001^‡^
Model 2	0.15	0.01^‡^	0.25	<0.001^‡^
Model 3	0.15	0.01^‡^	0.25	<0.001^‡^

Model 1: Unadjusted. Model 2: Linear regression adjusted for Age, ASMI, and BMI. Model 3: Linear regression adjusted for age, ASMI, BMI, exercise habits, and income.

*P < 0.05;^‡^*P* < (0.05/16).

BMI, body mass index; ASMI, appendicular skeletal muscle index.

## Discussion

This study investigated the association between the sex-based difference between CC and physical performance (hand grip strength and gait speed). Real-world information from an assessment of a community in northern Taiwan was collected. Low CC was found to be independently associated with poor physical performance, even after adjusting for age, BMI, and ASMI, especially in middle-aged and older people in Northern Taiwan. This is the first report in Taiwan to use a cross-sectional study to evaluate the sex-based differences between CC and physical performance, specifically among middle-aged and older people.

### Potential confounding factors for calf circumference

In this study, male participants were divided into three tertiles (<34.50, 34.50–36.75, and >36.75 cm), and female participants were distributed into three tertiles (<33.20, 33.20–35.50, and >35.50 cm). In contrast, the AWGS 2019 Consensus reported that using CC with threshold values of <34 cm in men and <33 cm in women could facilitate the earlier identification of people at risk of sarcopenia. The lower tertile of the present study approximates the threshold value of the AWGS 2019 consensus (6). According to Tables 2A,B, the CC tertile system was chosen due to the significant gait speed difference found under the tertile system in both sexes (*p* = 0.04), while the statistical difference in gait speed was observed only in women (*p* = 0.03 in women vs. *p* = 0.30 in men) using the binary splits system in the AWGS 2019 consensus. For both sexes, the average muscle strength and physical performance of the participants' CC in the lower tertile were mostly above the sarcopenia cutoff value of the AWGS 2019 consensus. Thus, CC may be a good screening tool for those with high sensitivity for sarcopenia screening in this study population, including those with poor physical performance. In both sexes, higher age, lower ASMI, lower hand grip strength, and lower gait speed were significantly associated with lower CC. Based on previous studies, aging may serve as an important part of the consequences of sarcopenia (26). Lower ASMI, hand grip strength, and gait speed are related to loss of muscle mass and physical performance in sarcopenia.

According to Tables 2A,B, the CC tertile system demonstrated potential confounding factors with sex-based differences in some of the general characteristics. Male participants with lower income and exercise habits had a higher proportion of CC <34.5 cm. One systematic review and meta-analysis showed that exercise has protective effects in older adults with sarcopenia (26). However, for women in our study, exercise appeared to be trending toward significance with CC tertiles (*p* = 0.06), and there may be a mild statistical aberration. Studies on the potential relationship between sarcopenia-related social factors (e.g., income and marital status) and sarcopenia remain limited. A systematic review and meta-analysis with a minimum recruitment age of 60, 65, and 70 years reported that the prevalence of frailty and pre-frailty appears higher in community-dwelling older adults in upper-middle-income economies [Gross National Income (GNI) per capita US$3,956–US$12,235 in 2017] than in high-income countries (GNI per capita > US$12,235 in 2017) (27). It has been postulated that a lower income affects nutritional and exercise resources, which may result in a greater risk of sarcopenia and frailty. Chinese male sex role attitudes may explain the sex income gap. Men are more likely to be the main source of household income (28). This may explain the sex-based difference in the significant correlation between CC increment and income, which showed no significance in females. In this study, female participants with underlying HTN were associated with CC tertiles. It is postulated that sarcopenia in female participants with HTN may be well-controlled by hypertensive medication, which potentially concealed their blood pressure measurement and questionnaire survey (participants may deny HTN due to being under control).

### Calf circumference as an independent predictor for physical performance

CC has been implemented as a screening tool in several definitions of sarcopenia (AWGS, EWGSOP, FNIH, IWGS, etc.). Sarcopenia and frailty diagnostic criteria typically include poor muscle strength and physical performance indices. A cross-sectional study showed that CC alone had the highest accuracy in a prediction model for sarcopenia compared to SARC-F, the 5-item Mini Sarcopenia Risk Assessment (MSRA-5), and SARC-CalF among residents over 65 years of age residing in two assisted living facilities in northern Taiwan (23). CC was associated with fat-free mass (β = 0.46, *p* = 0.01), skeletal muscle mass (β = 0.47, *p* = 0.01), and skeletal muscle mass index (β = 0.50, *p* = 0.01) in Netherlands's geriatric outpatients (average age: 80 years) (29). In another study, lower CC was an independent factor for fall incidence after adjusting for confounders, including demographics with age, sex, education, comorbidities, and functional/balance assessment among Peruvian older male adults aged 77.71 ± 8.55 from the Geriatrics Service at Peruvian Naval Medical Center (30). A lower CC was proposed to be an independent factor for physical performance [Short Physical Performance Battery (SPPB) score: 7.27 vs. 6.18, *p* = 0.02] and muscle strength (hand grip: 32 vs. 28 kg, *p* = 0.03) measures even after adjustment for potential confounders, including age, sex, education, body mass index, sensory impairments, cerebrovascular diseases, albumin, C-reactive protein, interleukin-6, and cholesterol, based on a study in a population of persons aged ≥80 years enrolled in Sirente, Italy (31). CC has also been reported to be an independent factor of frailty after adjusting for confounders such as age, sex, anthropometric indicators (BMI, WC, and HGS), and biological markers (hemoglobin, albumin, creatinine, and HbA1c) among Chinese patients aged ≥80 years with diabetes (32). Thus, lower CC may be an independent risk factor for poor physical performance in several ethnicities.

### Sex differences could be related to muscular integrity

Several other studies examining correlations between anthropometric indices and poor physical performance have studied sex differences. For instance, a cross-sectional cohort study analyzed data from 2,385 older participants (aged 70–84, 1,131 men and 1,254 women) in Korea. Poor physical performance in women (defined by SPPB) is correlated with fat-related body composition (body fat percentage, fat-mass index, and trunk fat-mass index), while better physical performance in men is associated with fat-free mass (18). In the present study, a lower CC was associated with worse hand grip strength and gait speed in both sexes. However, after adjusting for confounders with ASMI and BMI, CC was not associated with physical performance in men. Muscle mass in men may be another critical factor for physical performance compared to women.

Additionally, sex differences in skeletal muscle integrity may be a potential confounding factor. In previous studies, Haizlip et al. showed the prevalence of slower type-I and type-IIA fibers in women compared with men, which parallels the lower contractile velocity in women than in men (33). Fournier et al. found sex differences in semitendinosus muscle composition as a faster phenotype (65.8 ± 10.1 vs. 54.8 ± 8.3%, *p* < 0.05) and larger muscle size (myosin heavy chain (MyHC)-I, MyHC-IIA, and MyHC-IIX muscle fibers were 31, 43, and 50% larger) in men than in women by examining biopsy samples obtained from the semitendinosus muscle of 12 men and 12 women during anterior cruciate ligament reconstruction (34). However, the correlations between functional consequences and differences in muscular integrity should be further studied.

### Concerns about calf circumference screening

Some studies raised doubts about the correlation between CC and physical performance. A study cohort including 572 consecutive patients who were referred to geriatric outpatient clinics in Amsterdam showed that the association between CC and physical function measures [handgrip strength, SPPB, and the Timed Up and Go test (TUG)] was weak (29). CC may not be widely applicable for the estimation of physical performance in certain geriatric outpatient people. In addition, age cutoff values may vary with different ethnicities; therefore, further studies should be considered. Some researchers pointed out that CC may be overestimated owing to increased subcutaneous fat mass, which could hinder screening accuracy. However, a study in Japan recruited non-obese, healthy young adults and evaluated the thickness of the calf subcutaneous fat tissue and calf muscles using ultrasonic diagnostic equipment. When CC increases, calf subcutaneous fat tissue thickness should be reduced to maintain an equal percentage of body fat in young, healthy, and non-obese participants (35). This implies that subcutaneous fat thickness may not interfere with CC measurement, especially in non-obese participants. However, further studies on non-obese, middle-aged, and older people are required.

### Study limitations

This is the first cross-sectional study to investigate the association between sex-based differences in CC and physical performance in middle-aged, older Taiwanese individuals. However, this study had some limitations. First, the study was observational, and therefore, we were unable to describe the mechanism and causal relationship between physical performance and poor CC. Second, the generalizability of the findings to other people is not clear since the data collection was based on community-based participants only from northern Taiwan and included the possibility of healthy volunteer bias. Therefore, our study's results may not apply to other regions of Taiwan. A potential healthy volunteer bias might have lowered the prevalence of poor physical performance status and decreased our ability to control for confounding factors. Third, ~70% of the participants were women, which would have affected the male counterpart's CC tertile significance after adjusting for confounding factors. Fourth, the number of geriatric participants aged >80 is relatively limited. Finally, poor CC might be increased not only by the chronic medical conditions mentioned previously (DM, HTN, and hyperlipidemia) but also by diseases such as neurologic, rheumatologic, or other metabolic disorders, which could confound our results. More participants with multiple chronic conditions should be enrolled in future studies.

### Practical applications

The findings of this study suggest that CC tends to be an efficient predictor not only for sarcopenia risk but also for women's physical performance (hand grip strength and gait speed) in community health screenings and outpatient clinics for community health examinations, where survey facilities are limited.

## Conclusion

In conclusion, CC has the potential to be a useful measure of physical performance, especially in community-dwelling middle-aged, older women in Taiwan.

## Data availability statement

The raw data supporting the conclusions of this article will be made available by the authors, without undue reservation.

## Ethics statement

The studies involving human participants were reviewed and approved by Chang-Gung Medical Foundation Institutional Review Board. The patients/participants provided their written informed consent to participate in this study.

## Author contributions

Conceptualization, methodology, formal analysis, investigation, resources, project administration, and funding acquisition: J-YC. Writing—original draft preparation: P-CW. Writing—review and editing: P-CW and J-YC. Supervision: W-CY, Y-WT, and J-YC. All authors have read and agreed to the published version of the manuscript.
